# Mpox Panic, Infodemic, and Stigmatization of the Two-Spirit, Lesbian, Gay, Bisexual, Transgender, Queer or Questioning, Intersex, Asexual Community: Geospatial Analysis, Topic Modeling, and Sentiment Analysis of a Large, Multilingual Social Media Database

**DOI:** 10.2196/45108

**Published:** 2023-05-01

**Authors:** Zahra Movahedi Nia, Nicola Bragazzi, Ali Asgary, James Orbinski, Jianhong Wu, Jude Kong

**Affiliations:** 1 Africa-Canada Artificial Intelligence and Data Innovation Consortium, York University North York, ON Canada; 2 Laboratory for Industrial and Applied Mathematics, York University North York, ON Canada; 3 Advanced Disaster, Emergency and Rapid-response Simulation, York University North York, ON Canada; 4 Dahdaleh Institute for Global Health Research, York University North York, ON Canada

**Keywords:** monkeypox, infectious outbreak, infodemic, stigma, natural language processing, sentiment analysis, Twitter, community, discrimination, social media, virus

## Abstract

**Background:**

The global Mpox (formerly, Monkeypox) outbreak is disproportionately affecting the gay and bisexual men having sex with men community.

**Objective:**

The aim of this study is to use social media to study country-level variations in topics and sentiments toward Mpox and Two-Spirit, Lesbian, Gay, Bisexual, Transgender, Queer or Questioning, Intersex, Asexual (2SLGBTQIAP+)–related topics. Previous infectious outbreaks have shown that stigma intensifies an outbreak. This work helps health officials control fear and stop discrimination.

**Methods:**

In total, 125,424 Twitter and Facebook posts related to Mpox and the 2SLGBTQIAP+ community were extracted from May 1 to December 25, 2022, using Twitter application programming interface academic accounts and Facebook-scraper tools. The tweets’ main topics were discovered using Latent Dirichlet Allocation in the sklearn library. The *pysentimiento* package was used to find the sentiments of English and Spanish posts, and the *CamemBERT* package was used to recognize the sentiments of French posts. The tweets’ and Facebook posts’ languages were understood using the Twitter application programming interface platform and pycld3 library, respectively. Using ArcGis Online, the hot spots of the geotagged tweets were identified. Mann-Whitney *U*, ANOVA, and Dunn tests were used to compare the sentiment polarity of different topics and countries.

**Results:**

The number of Mpox posts and the number of posts with Mpox and 2SLGBTQIAP+ keywords were 85% correlated (*P*<.001). Interestingly, the number of posts with Mpox and 2SLGBTQIAP+ keywords had a higher correlation with the number of Mpox cases (correlation=0.36, *P*<.001) than the number of posts on Mpox (correlation=0.24, *P*<.001). Of the 10 topics, 8 were aimed at stigmatizing the 2SLGBTQIAP+ community, 3 of which had a significantly lower sentiment score than other topics (ANOVA *P*<.001). The Mann-Whitney *U* test shows that negative sentiments have a lower intensity than neutral and positive sentiments (*P*<.001) and neutral sentiments have a lower intensity than positive sentiments (*P*<.001). In addition, English sentiments have a higher negative and lower neutral and positive intensities than Spanish and French sentiments (*P*<.001), and Spanish sentiments have a higher negative and lower positive intensities than French sentiments (*P*<.001). The hot spots of the tweets with Mpox and 2SLGBTQIAP+ keywords were recognized as the United States, the United Kingdom, Canada, Spain, Portugal, India, Ireland, and Italy. Canada was identified as having more tweets with negative polarity and a lower sentiment score (*P*<.04).

**Conclusions:**

The 2SLGBTQIAP+ community is being widely stigmatized for spreading the Mpox virus on social media. This turns the community into a highly vulnerable population, widens the disparities, increases discrimination, and accelerates the spread of the virus. By identifying the hot spots and key topics of the related tweets, this work helps decision makers and health officials inform more targeted policies.

## Introduction

While smallpox, an infection caused by the Variola virus (Poxviridae family, Chordopoxvirinae subfamily, and *Orthopoxvirus* genus), has been declared fully eradicated in 1980 [1], Mpox (formerly, Monkeypox), a similar but milder communicable disorder caused by another *Orthopoxvirus* (Monkeypox virus, MPXV), is still circulating [2,3]. This disease has been endemic in a number of African countries since 1970, when the first known human case of Mpox was reported in a 9-month-old child [3], after being isolated in 1958 [3]. A complex interplay of various variables, including geographical, environmental, historical, and socioeconomic factors such as political instability, population mobility, and poverty, as well as the waning of the immunity conferred by vaccinia vaccination, may have contributed to its spreading in Africa [3-6]. Two major MPXV clades have been discovered and identified, namely, the Central African (Congo Basin) Clade, now referred to as Clade one (I), and the Western African Clade, renamed Clade two (II) [7], consisting of 2 subclades (Clade IIa and Clade IIb) [8]. The ecoregion extending from southeastern Nigeria to Cameroon, between the Cross and Sanaga rivers, may have acted as a biogeographic barrier, splitting the virus into these 2 genomic variants [3,7-9].

Until 2003, Mpox had never been reported outside of African countries, being, as previously mentioned, endemic in West and Central Africa. In 2003, a cluster of 47 cases was announced in the United States, linked to imported African small mammals [10]. Since then, Mpox outbreaks have been reported in non-African countries. Israel reported its first case in 2018 [11]. The United Kingdom reported 2 major clusters in 2018 and 1 in 2019 [12,13], and Singapore announced its first case in 2019 [14].

The ongoing global Mpox outbreak started on May 6, 2022, in the United Kingdom, with one case being a British resident traveling back from Nigeria, but with other case clusters of unclear epidemiological origin [15]. Since then, an increasing number of countries have progressively announced new cases, with some hospitalizations [16] and a few fatalities [3].

As of November 10, 2022, according to the US Centers for Disease Control and Prevention (CDC), globally, 110 countries have been affected by Mpox cases, 103 of which have not historically reported Mpox [17], with Clade IIb being the MPXV viral strain currently circulating [8]. The current outbreak is characterized by an emerging transmission route, which is to say, it is spread by close physical or sexual contact [18]. Since 93%-98% [19,20] of reported Mpox cases affected gay and bisexual men having sex with men [21], public and global health responses have mainly targeted the Two-Spirit, Lesbian, Gay, Bisexual, Transgender, Queer or Questioning, Intersex, Asexual (2SLGBTQIAP+) community, which has been heavily and disproportionately impacted by the ongoing global outbreak [19,22]. With this particular focus on this specific population, despite the fact that the infectious agent can be spread and acquired through multiple pathways, the 2SLGBTQIAP+ community risks being stigmatized and blamed for transmitting the virus [19,22]. Stigmatization represents a serious public health concern in that, during infectious outbreaks, it can cause more new cases and aggravate underlying health vulnerabilities [22,23]. Past experiences have shown that blaming marginalized and minority populations for spreading the disease can increase the risk of individuals not seeking health care upon observing their symptoms due to the fear of stigma [24,25]. Moreover, stigma causes depression, mental health conditions, psychological damage, and increases substance use [26-28]. Previous works have studied the negative impact of stigmatizing minority groups and communities during various infectious outbreaks, for example, HIV/AIDS and hepatitis B and C [29-32]. For instance, Nyblade [29] conducted a survey to assess the impact of HIV/AIDS-related stigma and public opinion on the spread of the virus. The results showed that at the beginning, stigma contributed to fueling the virus transmission, with discrimination gradually decreasing and allowing more patients to seek help. Grossman and Stangl [30] described how to devise strategies and interventions aimed at reducing HIV/AIDS stigma and counteracting and mitigating its effects, whereas Shen et al [32] assessed the effectiveness of a crowdsourced intervention for decreasing hepatitis B–related stigma in the men having sex with men community.

As such, given its implications in terms of public and global health, it is of paramount importance to measure stigma. Currently, there exist various tools and techniques to do so; for instance, some authors [33] have designed a questionnaire using the EPI (CDC) software and built a stigma score. The results showed a direct connection between stigma and depression. They also found that stigma is different across various cultures and populations. Holzemer et al [34] had 726 patients infected with HIV from Africa, Puerto Rico, and the United States fill out a questionnaire designed for assessing the quality of life. The results showed that the patients were highly scared of stigma as a discrediting social label and were highly reluctant and hesitant to be tested. They displayed a low quality of life, and many of them endured depression. It was shown in [35] that stigmatization and marginalization of the minor population are associated with increased alcohol use. Furthermore, a study [36], based on questionnaires and focus group discussions, was conducted to analyze stigma among African American rural adolescents. The results showed that participants had an average level of HIV/AIDS knowledge and that stigma played a major role in the risk of contracting HIV and developing AIDS. Moreover, they found that nurses and other health care professionals can play a key role in addressing HIV/AIDS–related stigma and misconceptions among the lay population.

However, questionnaires, even though specifically designed for measuring stigma, have some major shortcomings. “Novel and unconventional data streams” [37,38], including social media and social networks, offer unprecedented opportunities to quantify the levels of stigma and track them in real time. Some authors [39] used the stigmatization term frequency of Twitter posts to assess stigma for different debilitating conditions affecting physical and mental health, including HIV/AIDS. The results showed that people with mental health conditions are more stigmatized than people with physical health issues, and, in turn, people living with HIV/AIDS are more stigmatized than people with mental health conditions. A survey [40] conducted among African-American and Latino men having sex with men showed that stigma was positively associated with the number of hours spent on social media. In other words, individuals that had a higher sense of stigma were more likely to spend their time on social media platforms such as Facebook. Veinot et al [41] conclude that reducing stigma improves information-seeking and sharing behavior, helps individuals have better access to information on sexually transmitted diseases, particularly HIV and AIDS, and decreases the risk of infection.

On the other hand, social media can also reduce stigma and mitigate discrimination [42,43]. In more detail, three ways to counteract stigma in social media have been identified [43], namely, (1) protest, (2) education, and (3) contact. Concerning the former way (protest), direct messages or hashtags opposing stigmatization, such as “Stop the Stigma,” can be propagated as much as possible. Concerning education, true, verified, and accurate information can be posted in response to false and misleading information. Concerning contact, a stigmatized individual comes into contact with a stigmatizing user in order for them to hear and understand the opposite side. However, before taking any action and implementing a solution, the stigma should be investigated in depth in terms of topics of interest and sentiments. natural language processing (NLP) techniques, like topic modeling and sentiment analysis [44], coupled with geospatial software, can help achieve this aim. Moreover, authors in [45-47] found that using web-based testing, message-based surveys, and mobile health interventions instead of in-person questionnaires reduces concern over privacy and stigmatization and helps marginalized populations share truthful information for better health services.

Specifically concerning Mpox, a few works have focused on the stigmatization of the 2SLGBTQIAP+ community during the 2022 global Mpox outbreak. Some authors [48], given the global burden imposed by stigma, have warned of the consequences of ignoring stigmatization as it happened during previous infectious outbreaks, such as HIV/AIDS, and advised public health officials to proactively address Mpox-related stigmatization. Other authors [49] have discussed guidelines for taking care of and helping minority communities, emphasizing the importance of raising awareness about the risk of Mpox and the implications of stigmatizing sexual and gender minority populations [50].

Even though the abovementioned studies provided rich information and insightful comments regarding the consequences of blaming minority populations for spreading Mpox, as well as suggestions for mitigating these issues, only a few of them have adopted a social media perspective [51-53]. Social media is a web-based environment where people can share their thoughts, ideas, and beliefs. Novel information, as well as fabricated and misleading information, and fake news, can be massively propagated using these platforms every day [54,55].

Previous works have exploited social media for various purposes, including opinion mining [56], hot spot detection [57], and surveillance [58]. In [59], 137 Tik Tok videos were manually screened and categorized. Roughly 12% of the videos came from the Lesbian, Gay, Bisexual, Transgender, Queer (LGBTQ) category. However, the stigmatization of the LGBTQ community was not studied. Dsouza et al [60] analyzed the sentiments of tweets to study the LGBTQ stigmatization for spreading Mpox. However, they only considered English tweets, did not perform cross-country analysis, and did not extract and analyze discussed topics on social media.

In this paper, we fill in the gap by studying social media in more depth and leveraging Twitter and Facebook to better understand Mpox-related stigmatization by assessing relevant popular discussions and conversations regarding Mpox, identifying stigmatization sources, their hot spots, and their sentiments. A data set was built and analyzed by gathering relevant posts from Twitter and Facebook and using keywords related to the Mpox. Two NLP techniques, namely, topic modeling and sentiment analysis, were performed on the posts. ArcGis Online [61,62] was used to visualize the geotagged tweets and find their hot spots. The result of our work may have practical implications in that it could be used by public health officials to determine the direction of their policies and inform them in a data-driven fashion.

## Methods

### Gathering the Data Set

The data set for this work was gathered from 2 of the most popular social media platforms: Twitter and Facebook. By using the full-archive search of the Twitter Academic Researcher Application Programming Interface, all the tweets posted since 2006 can be retrieved for a given query [63,64]. Using keywords related to Mpox, a query was built ((monkeypox OR “monkey pox” OR smallpox OR “viruela dei mono” OR “variole du singe” OR “variola do macaco”) -is:retweet) to gather all the tweets except the retweets, from May 1 to December 25, 2022. The tweets were cleaned. URLs, addresses with the “@” sign, and hashtag signs “#” were removed, and punctuations were corrected. Finally, 2,333,496 cleaned tweets were obtained. 124,712 tweets related to the 2SLGBTQIAP+ community were extracted using the following keywords: lgbtq, lgbtq+, gay, homosexual, homosexuality, lesbian, intersex, transsexual, transgender, bisexual, queer, “men having sex with men,” “men who have sex with men,” lgbt, lgbtqi, lgbt+, lgbtqi+, and lgbtq+ [65]. All the posts in 30 Mpox-related public Facebook groups from May 1 to December 25, 2022, were gathered using the Facebook_Scraper library [66]. Of the 16,114 retrieved posts, 712 had the 2SLGBTQIAP+ keywords after cleaning and were selected for analysis. All Facebook posts are public and gathered from public groups. The Twitter and Facebook data sets gathered with Mpox- and Mpox plus 2SLGBTQIAP+-related keywords or groups were combined and visualized by means of a word cloud (Multimedia Appendix 1).

The language of the tweets was retrieved using the Twitter application programming interface. Moreover, the language of Facebook posts was recognized using the pycld3 library [67]. The posts were in 102 different languages, and English, French, and Spanish posts with 1,972,637, 124,008, and 33,547 posts, respectively, had the highest frequency.

### Ethical Considerations

We have gathered public posts from public pages and public groups on Facebook, accessible by anyone through Facebook. We share the group IDs, page IDs, and post IDs of our data set [68], in compliance with the Association of Internet Researchers ethics [69] and the International Chamber of Commerce/European Society for Opinion and Marketing Research code of conduct [70]. Moreover, the Twitter data set, which is available at [71], includes only tweet IDs and user IDs and is used and shared under Twitter’s privacy policy agreement [72]. Since social media posts are passively analyzed in this research, informed consent from individuals is waived [73].

### NLP Techniques

Topic modeling, a text mining tool for automatic discovery and extraction of hidden topics and semantic structures occurring within a text body or in a collection of documents, was done using the Latent Dirichlet Allocation model available in the *sklearn* package of Python (version 3.8.8; Python Software Foundation). Topic analysis was performed only on English posts. The optimal number of topics was calculated by maximization of the coherence and minimization of the Jaccard similarity scores [74]. Posts belonging to each topic with a probability higher than 0.7 were studied, and the main subject of concern for each topic was inferred.

In this paper, topic modeling was coupled with sentiment analysis, which is an NLP procedure that classifies a text based on its sentiment. Most sentiment analysis models classify a text into 3 classes: positive, neutral, and negative. However, some models classify a text into 2 classes: positive and negative. The sentiment score is a number between –1 and 1, which indicates the intensity of the sentiment. Generally, models that classify a text into 3 classes have a score close to 1, 0, and –1 for positive, neutral, and negative sentiments, respectively. Moreover, models that classify a text into 2 classes provide a negative score for negative sentiments and a positive score for positive sentiments. In this work, sentiment analysis was performed on English and Spanish posts using the *pysentimiento* package available on the Hugging Face website. It is estimated that this model, which classifies text into 3 classes, has a macro *F*_1_ score of 0.705 [75-78]. Sentiment analysis was performed on French posts using CamemBERT, which classifies text into 2 classes and is estimated to have 94.55% accuracy [79]. Geotagged tweets were used to study topics and sentiments regarding the 2SLGBTQIAP+ community across different countries. Sentiments on different topics were compared using Mann-Whitney *U*, ANOVA, and Dunn tests, and studied across different countries using the Mann-Whitney *U* test.

## Results

### Trends in the Posts

**Figure 1:**
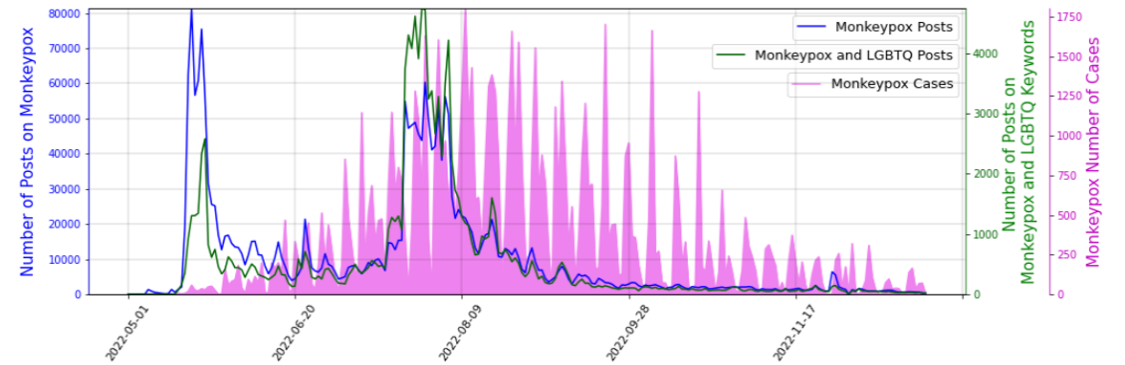
The trend of posts on Mpox and Mpox plus Two-Spirit, Lesbian, Gay, Bisexual, Transgender, Queer and/or Questioning, Intersex, Asexual (2SLGBTQIAP+) keywords and the number of Mpox cases. LGBTQ: Lesbian, Gay, Bisexual, Transgender, Queer.

The temporal trend of the number of the gathered posts related to Mpox and its epidemiology in terms of Mpox cases is depicted in Figure 1. From May 18, when Mpox began to emerge as an outbreak, the volume of the posts significantly increased until May 24, when they started falling. Since May 28, the volume of posts has stayed more or less steady. In more detail, the number of Mpox posts peaked on May 20 and on May 23, 2022, while the number of Mpox and the 2SLGBTQIAP+ community posts peaked on May 24, 2022, just 2 days after the Joint United Nations Programme on HIV and AIDS (ie, UNAIDS) urged media outlets, as well as institutional actors, including governments and communities, to respond to the outbreak with an evidence-based, data-driven, and, at the same time, inclusive and rights-based approach, avoiding attaching a stigma to the 2SLGBTQIAP+ community.

The number of posts concerning the Mpox and those specifically focusing on the relationship between the Mpox and the 2SLGBTQIAP+ community were highly correlated, as expected (correlation coefficient of 0.85, *P*<.001). Interestingly, the correlation between the number of Mpox cases and the number of posts related to the 2SLGBTQIAP+ community (correlation coefficient of 0.36, *P*<.001) was higher than the correlation between the Mpox cases and the total number of posts on Mpox (correlation coefficient of 0.24, *P*<.001). This shows how closely the discussions regarding Mpox on social media are related to the 2SLGBTQIAP+ community.

### Topic Modeling

A total of 10 different topics were extracted from posts related to Mpox and 2SLGBTQIAP+. The topics indicate that the 2SLGBTQIAP+ population is heavily stigmatized for spreading Mpox. Table S1 in Multimedia Appendix 2 shows the identified keywords and the percentage of tweets on each topic that have them. The first 17 keywords shaded in gray are essentially related to Mpox and 2SLGBTQIAP+ and are common among almost all of the topics. The rest of the keywords dominantly belong to one of the topics. The same pattern could be observed in the word clouds created for each topic (Multimedia Appendix 3). This indicates that the topics are well separated and do not overlap. This is the result of maximizing coherence while minimizing the Jaccard similarity score. In other words, posts inside each topic are very much related, and posts from different topics are far from each other. By studying the posts that belong to each topic with a probability higher than 0.7, the subjects identified for each topic listed in Textbox 1.

**Identified subjects.**

On studying the posts belonging to each topic (probability>0.7), the subjects identified for each topic are as follows:

- Topic #1: lesbian, gay, bisexual, transgender, queer pride
- Topic #2: What World Health Organization/public health/health officials say about Mpox; Mpox is/is not a gay disease
- Topic #3: Mpox does/does not spread through gays/gay orgies/queers
- Topic #4: Mpox is an airborne bioweapon targeting gays
- Topic #5: Reporting number of cases in different countries; Condition of having rash or lesion on skin
- Topic #6: gay bathhouse/homosexuality/heterosexual; Centers for Disease Control and Prevention and CNN (Cable News Network) news.
- Topic #7: Mpox spreads through gay/homosexual sex
- Topic #8: Mpox outbreak linked to gay sauna/gay bars/Grindr/fetish festival; Avoid gay sex to protect yourself
- Topic #9: Mpox is a stigma against gays/African gays; stigmatizing gays/African gays
- Topic #10: Mpox particularly concentrates on gay and bisexual men, however, anyone could be at risk

### Sentiment Analysis

**Figure 2:**
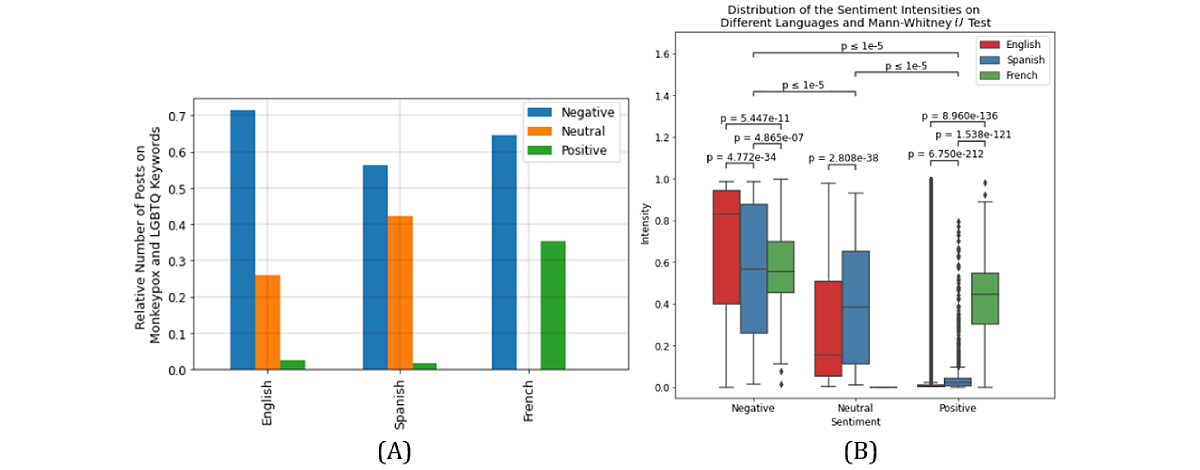
(A) Relative number and (B) sentiment intensity of posts gathered with Mpox and LGBTQ keywords for each polarity for English, Spanish, and French posts. LGBTQ: Lesbian, Gay, Bisexual, Transgender, Queer.

Topic modeling indicated that the 2SLGBTQIAP+ population is highly stigmatized for spreading Mpox. Sentiment analysis shows that most sentiments are negative, then neutral, and only a few are positive. Since the posts are related to an outbreak after a pandemic and the stigmatization of minor populations, it is expected that the sentiments be mostly negative. English posts have the greatest number of negative polarities. Spanish posts have the fewest negative polarities, and a higher neutral polarity compared to English posts. Moreover, the negative polarity of French posts is significantly higher than the positive polarity (Figure 2). The *P* value of the Mann-Whitney *U* test indicates that the intensity of negative polarity is significantly higher than that of neutral and positive polarities (*P*<.001), and the intensity of neutral polarity is significantly higher than that of positive polarity (*P*<.001; Figure 2). The *P* value of the Mann-Whitney *U* test also indicates that English posts have a significantly higher negative intensity and lower neutral and positive intensities compared to Spanish and French posts (*P*<.001; Figure 2).

**Figure 3:**
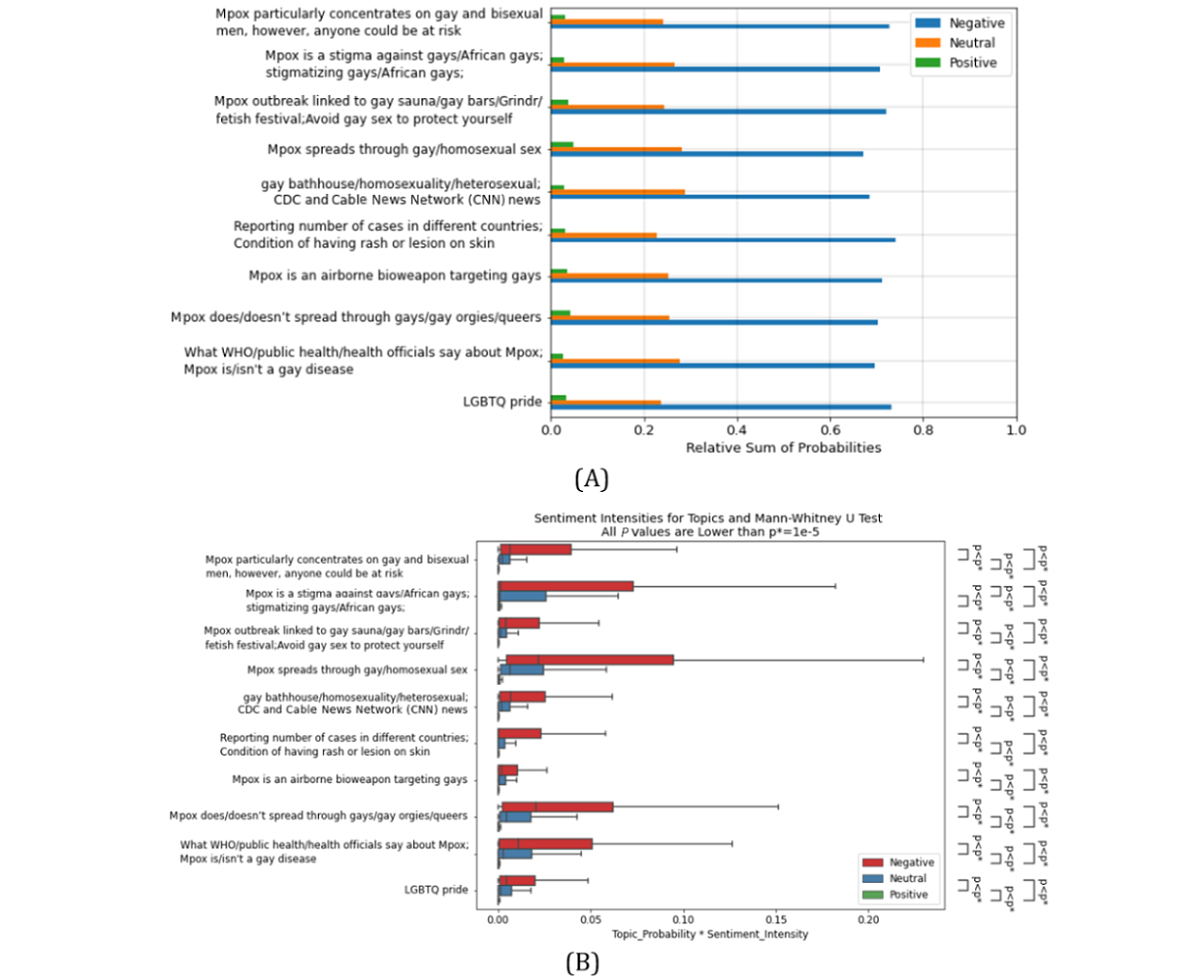
(A) Sentiment polarity and (B) sentiment intensity on different topics. CDC: Centers for Disease Control and Prevention; LGBTQ: Lesbian, Gay, Bisexual, Transgender, Queer; WHO: World Health Organization.

**Figure 4:**
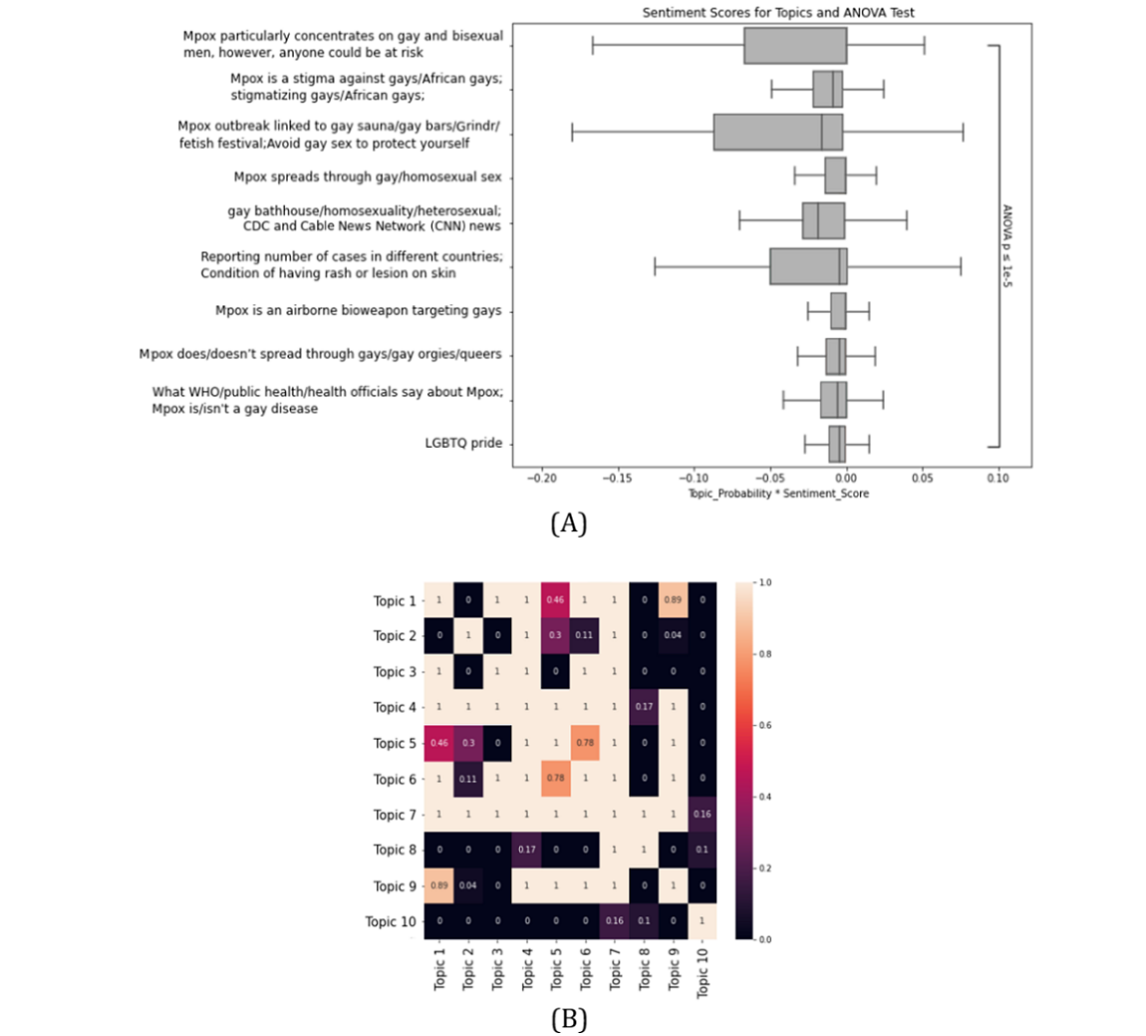
(A) Distribution of sentiment scores on different topics and ANOVA test. (B) Recognizing topics with lower sentiment scores with Dunn test. CDC: Centers for Disease Control and Prevention; LGBTQ: Lesbian, Gay, Bisexual, Transgender, Queer; WHO: World Health Organization.

All the topics have a higher negative, then neutral, and finally positive polarity (Figure 3). Additionally, the Mann-Whitney *U* test shows that all the topics have significantly higher negative, then neutral, and finally positive intensities (Figure 3). However, the ANOVA test indicates that the sentiment scores of different topics are not very similar (*P*<.001; Figure 4). The Dunn test shows that 3 topics that are strongly related to the stigmatization of the 2SLGBTQIAP+ community, namely, “gay bathhouse/homosexuality/heterosexual; CDC and CNN news,” “Mpox outbreak linked to gay sauna/gay bars/Grindr/fetish festival; Avoid gay sex to protect yourself,” and “Mpox is a stigma against gays/African gays; Stigmatizing gays/African gays” have a significantly lower sentiment score compared to other topics (Figure 4).

### Hot Spots

**Figure 5:**
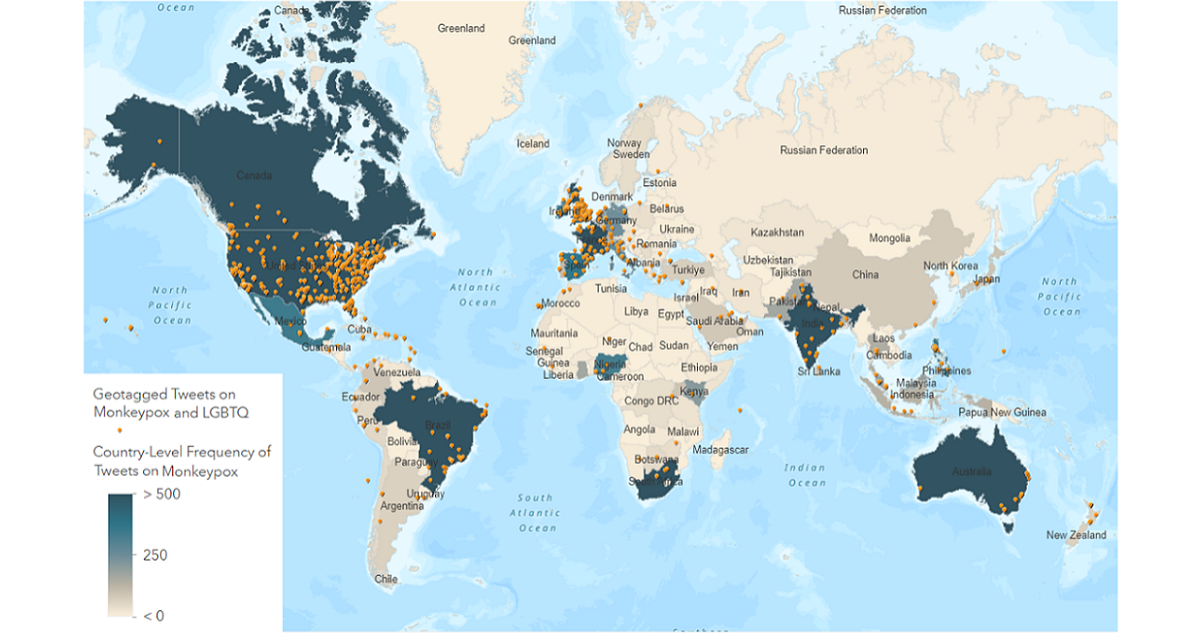
Visualization of geotagged tweets on Mpox and Mpox plus LGBTQ keywords. LGBTQ: Lesbian, Gay, Bisexual, Transgender, Queer.

The visualization of the geotagged tweets gathered on Mpox and Mpox plus LGBTQ keywords shows that countries that have the greatest number of tweets include the United States, the United Kingdom, Canada, Ireland, France, the Netherlands, Switzerland, Spain, Portugal, Germany, Mexico, Brazil, South Africa, Nigeria, Kenya, Pakistan, and India (Figure 5). The tweets extracted for the 2SLGBTQIAP+ community were mostly concentrated in the United States, the United Kingdom, Canada, Spain, Portugal, India, Ireland, and Italy. However, topic modeling of tweets related to Mpox and the 2SLGBTQIAP+ community was performed only on English tweets, which were mainly from the United States, the United Kingdom, Canada, and India.

**Figure 6:**
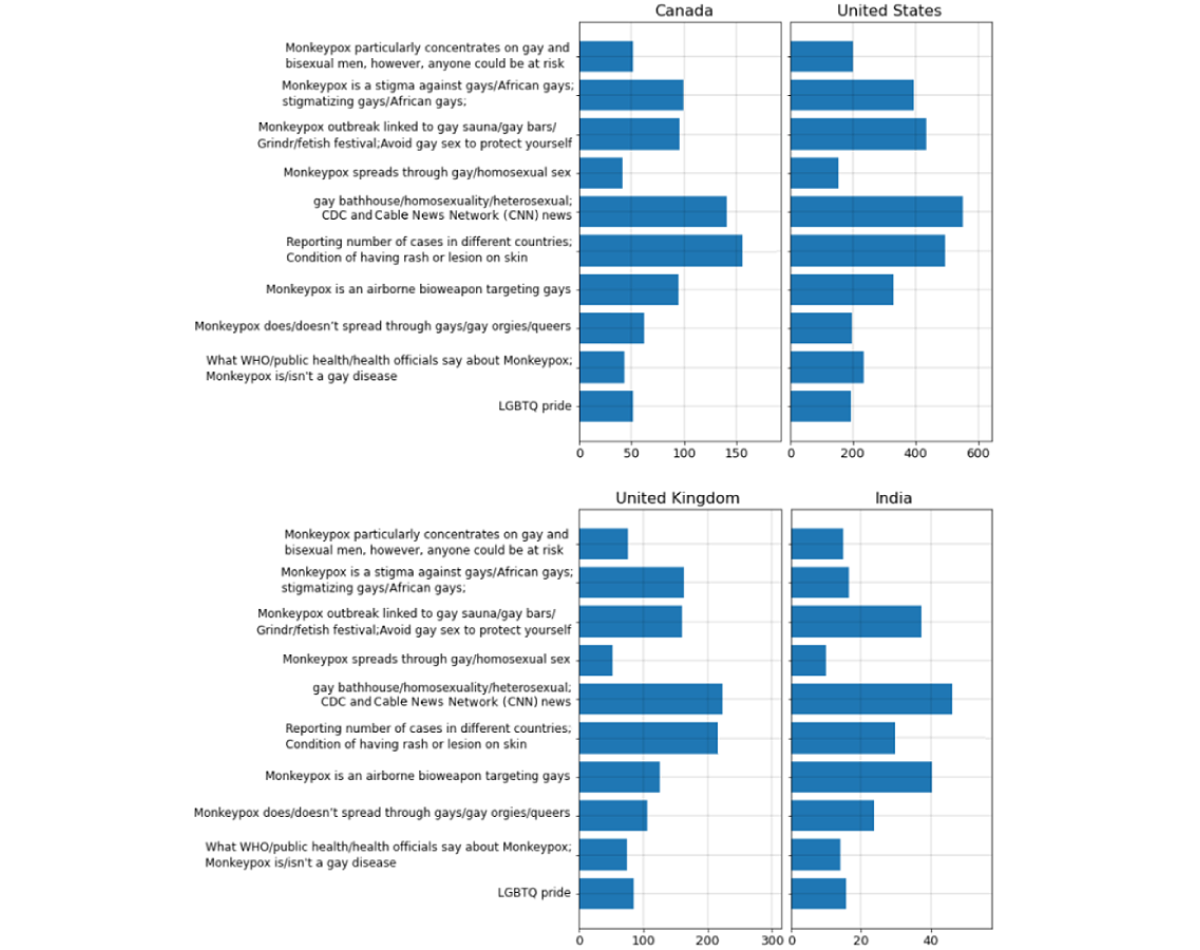
Tweets belonging to each topic for Mpox and the Two-Spirit, Lesbian, Gay, Bisexual, Transgender, Queer and/or Questioning, Intersex, Asexual (2SLGBTQIAP+) community in different countries. CDC: Centers for Disease Control and Prevention; LGBTQ: Lesbian, Gay, Bisexual, Transgender, Queer; WHO: World Health Organization.

After topic numbers 5 and 6, which are about the news and reporting the number of Mpox cases, topics 3, 4, 8, and 9 have the most popularity among different countries, which are all related to the 2SLGBTQIAP+ population being stigmatized for spreading Mpox (Figure 6). Since the posts were in English, it was possible to find the popularity of each topic only in 4 different countries: the United States, the United Kingdom, Canada, and India.

**Figure 7:**
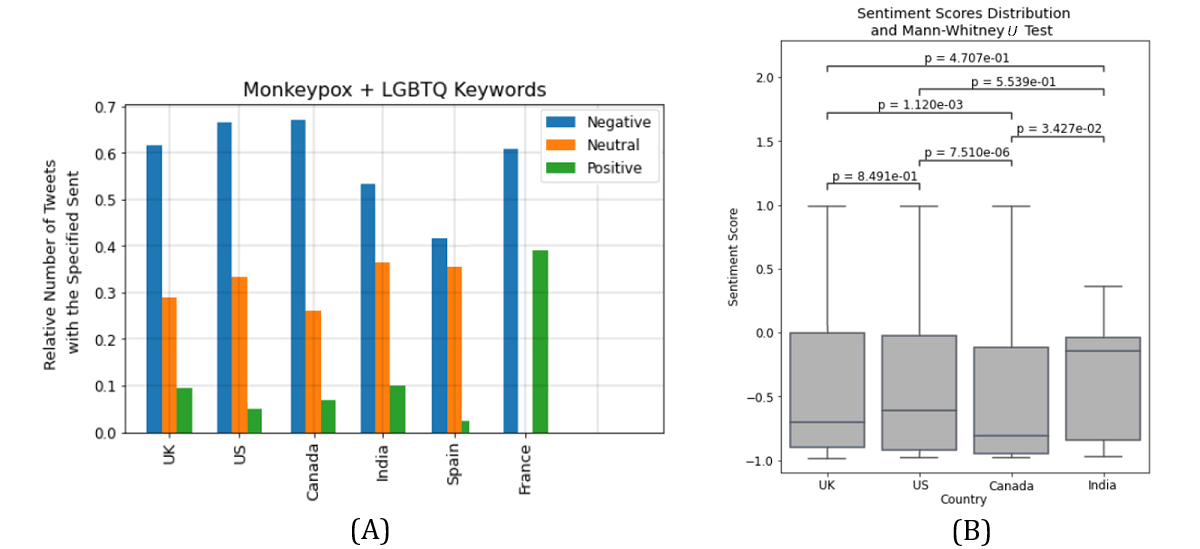
(A) Sentiment polarity in different countries. (B) Distribution of sentiment scores across different countries and Mann-Whitney *U* test. LGBTQ: Lesbian, Gay, Bisexual, Transgender, Queer; UK: United Kingdom; US: United States.

Sentiment polarities were found for English tweets in the United States, the United Kingdom, Canada, and India. Moreover, the sentiment polarities of the Spanish and French tweets were found only for Spain and France, respectively, since the volume was low for other countries. The 3 countries with the highest negative polarity are Canada, the United States, and the United Kingdom (Figure 7). The *P* value of the Mann-Whitney *U* test indicates that the distribution of sentiment scores across different countries is less diverse (Figure 7). However, among the countries that were studied for English tweets, Canada has the lowest sentiment score (*P*<.04).

## Discussion

### Principal Findings

On July 23, 2022, the World Health Organization (WHO) declared Mpox a public health emergency of international concern [80]. Ever since then, the number of cases around the globe has been increasing. Previously, minority populations such as gay and bisexual communities were blamed for spreading different diseases like HIV/AIDS and hepatitis B and C. The result of such stigmatization was more new cases, depression, mental health problems, and substance use [81]. The same trend is observable with the novel Mpox outbreak that is spreading around the world. This work aims to understand Mpox stigmatization of the 2SLGBTQIAP+ community using Twitter and Facebook.

Social media is becoming increasingly popular among people to share their opinions, ideas, and experiences. People are sometimes more honest on social media than in their real lives. Therefore, it is a reflection of the real world. As a result, it is used in many different areas of research, such as the economy [82], marketing [83], and health care [84].

A few studies have applied social media mining to Mpox. Ng et al [52] extracted from Twitter a body of 352,182 original tweets containing the terms “monkeypox,” “monkey pox,” or “monkey_pox,” in the English language, from May 6, 2022, to July 23, 2022, using Bidirectional Encoder Representations from Transformers named entity recognition. The authors identified 5 topics clustered into three major themes: (1) safety concerns, (2) sexual and gender minority stigmatization, and (3) a general lack of faith in public institutions. Tweets displayed high levels of partisanship and personal health anxiety.

In line with these findings, in this paper, Tweets and Facebook posts are used to discover the popular discussions regarding Mpox and the stigmatization of 2SLGBTQIAP+ communities, the hot spots, and the sentiments of different topics and countries. The results of this study could be used by health officials to combat stigmatization.

### Strengths and Limitations

This investigation has a number of strengths, including its methodological rigor, transparency, and novelty, as well as its main focus on the 2SLGBTQIAP+ community. On the other hand, it is not without any limitations, which should be properly acknowledged. Sentiments analysis was performed on English, French, and Spanish tweets and Facebook posts, which had the highest frequency among all the different languages. Moreover, topic modeling was performed on English posts, which made up more than 81% of the posts. As a result, the discussions and sentiments of the majority of the posts have been extracted and analyzed. However, there are countries in which people do not speak English, French, or Spanish. The analysis of this study could not be generalized to the countries whose official languages are different.

### Conclusions

The number of posts with Mpox and 2SLGBTQIAP+ keywords had a higher correlation with the number of Mpox cases (correlation coefficient of 0.36, *P*<.001) compared to the number of posts on Mpox (correlation coefficient of 0.24, *P*<.001). This indicates that social media discussions on Mpox are tightly related to the 2SLGBTQIAP+ community. Out of the 10 topics related to Mpox and LGBTQ, 8 were directly focused on blaming the gay community for spreading Mpox. The sentiments on all topics were very negative. Three of the topics that were strongly related to the stigmatization of the 2SLGBTQIAP+ community had a significantly lower sentiment score compared to other topics (ANOVA *P*<.001). The sentiment of posts from all the 3 languages, English, Spanish, and French, had a higher negative intensity, then neutral, and then positive (*P*<.001). Canada had the lowest sentiment score compared to other countries (*P*<.04). Stigmatization of a minority community on this scale will cause seclusion of people and increase hesitancy for seeking help upon realization of the symptoms. Stigmatization of the gay community, especially in countries where the sentiment polarity is very negative (ie, Canada, the United States, and the United Kingdom), must be prevented in order to contain Mpox and control the disease.

As a contribution to the future of this work, NLP tools could be used to study the sentiments and topics of posts regarding Mpox and the 2SLGBTQIAP+ community in languages and regions other than the ones studied in this manuscript.

## Appendix

Multimedia Appendix 1 The Twitter and Facebook data sets with (A) Mpox and (B) Mpox plus Two-Spirit, Lesbian, Gay, Bisexual, Transgender, Queer and/or Questioning, Intersex, Asexual (2SLGBTQIAP+) keywords and groups shown by word cloud.

Multimedia Appendix 2 The most prominent keywords of each topic and the percentage of their contribution in building that topic for posts on Mpox plus 2SLGBTQIAP+.

Multimedia Appendix 3 Visualizing each topic using a word cloud.

## Abbreviations

2SLGBTQIAP+: Two-Spirit, Lesbian, Gay, Bisexual, Transgender, Queer and/or Questioning, Intersex, Asexual
CDC: Centers for Disease Control and Prevention
LGBTQ: Lesbian, Gay, Bisexual, Transgender, Queer
MPXV: Monkeypox virus
NLP: natural language processing

## Data Availability

The Facebook data set generated during or analyzed during this study is available on GitHub [68]. The Twitter data set generated during or analyzed during this study is available from Movahedi et al [71].
