# Supplementary figures and images for: Mpox Panic, Infodemic, and Stigmatization of the Two-Spirit, Lesbian, Gay, Bisexual, Transgender, Queer or Questioning, Intersex, Asexual Community: Geospatial Analysis, Topic Modeling, and Sentiment Analysis of a Large, Multilingual Social Media Database
Source: J Med Internet Res. 2023 May 1;25:e45108. doi: 10.2196/45108 (PMC10186192; doi:10.2196/45108)

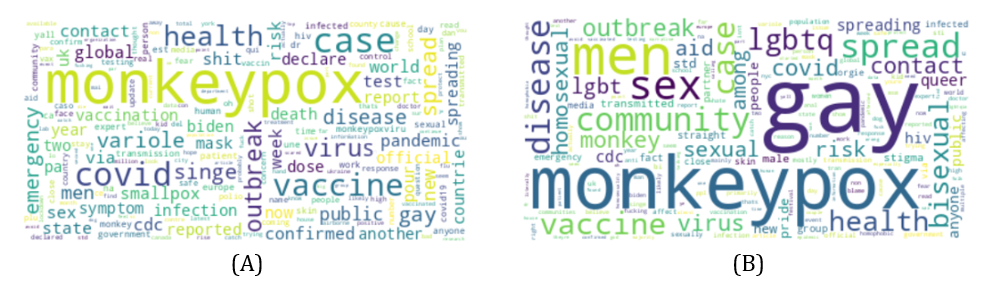

Supplement: Multimedia Appendix 1 [file jmir_v25i1e45108_app1.png]

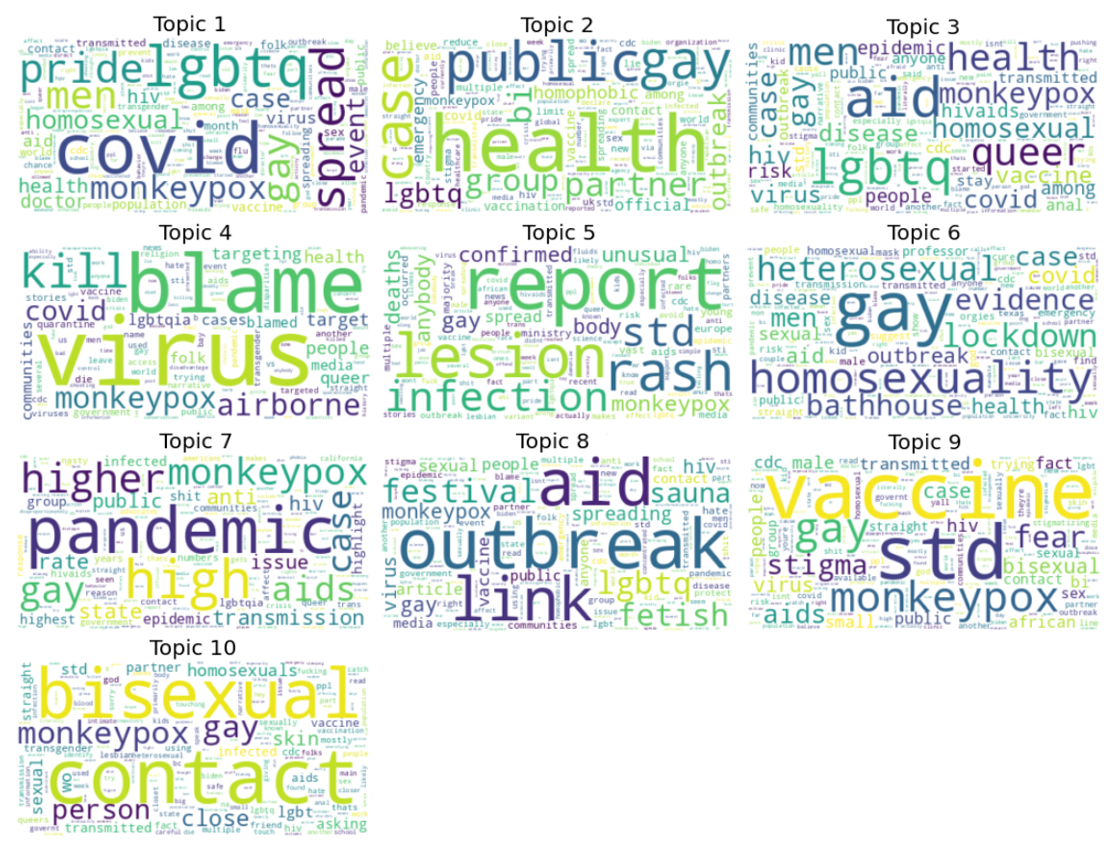

Supplement: Multimedia Appendix 3 [file jmir_v25i1e45108_app3.png]
