# Supplementary material for: Mpox Panic, Infodemic, and Stigmatization of the Two-Spirit, Lesbian, Gay, Bisexual, Transgender, Queer or Questioning, Intersex, Asexual Community: Geospatial Analysis, Topic Modeling, and Sentiment Analysis of a Large, Multilingual Social Media Database
Source: J Med Internet Res. 2023 May 1;25:e45108. doi: 10.2196/45108 (PMC10186192; doi:10.2196/45108)
Supplement: Multimedia Appendix 2 [file jmir_v25i1e45108_app2.pdf]

This is a Multimedia Appendix to a full manuscript entitled, "Mpox Panic, Infodemic, and Stigmatization of the 2SLGBTQIAP+ Community: Geospatial Analysis, Topic Modeling, and Sentiment Analysis of a Large, Multilingual Social Media Database" published in the J Med Internet Res. For full copyright and citation information see http://dx.doi.org/10.2196/45108.

Table 1 shows the most prominent keywords of each topic and the percentage of their contribution in building that topic.

Table 1. Keywords of different topics for posts on Mpox and the 2SLGBTQIAP+ (each column represents a different topic and each row is a keyword)

No		1	2	3	4	5	6	7	8	9	10
1	monkeypox	9%		8%	32%	9%		12%	16%	6%	8%
2	case					50%			10%		40%
3	spread			2%	40%		5%	9%		4%	40%
4	contact					25%					75%
5	virus		8%		9%		34%	17%			32%
6	outbreak				10%			3%	79%		8%
7	transmit							45%			
8	risk	15%	7%					21%	20%		37%
9	disease		21%		40%			24%			15%
10	gay	8%		6%	36%	9%		10%	16%		15%
11	man		7%		20%	15%		4%	3%		52%
12	sex				13%			8%			
13	bisexual					23%					77%
14	sexual					35%					

15	community	9%	18%		40%		28%			5%	
16	male			18%	12%			40%			30%
17	medium	2%	10%	80%				8%			
18	attend	100%									
19	event	95%	5%								
20	pride	80%			20%						
21	doctor	100%									
22	covid	100%									
23	superspreader	100%									
24	chance	100%									
25	low	88%		12%							
26	direct	100%									
27	warn	59%									41%
28	public		100%								
29	health		75%			25%					
30	official		100%								
31	authority		100%								
32	group		100%								

33	country	16%	84%								
34	government		100%								
35	lie		100%								
36	vaccination		100%								
37	label		100%								
38	prevalent		100%								
39	partner		100%								
40	homophobic		100%								
41	queer			100%							
42	orgy			100%							
43	racist			100%							
44	aid			100%							
45	anal			100%							
46	push			100%							
47	immune			100%							
48	viral			100%							
49	stay			100%							
50	rash					100%					
51	lesion					100%					

52	symptom		4%	1%		65%					30%
53	confirm					100%					
54	report					86%				14%	
55	body					100%					
56	rare					100%					
57	unusual					100%					
58	link								94%		6%
59	sauna								100%		
60	festival	8%			5%				85%		2%
61	lgbt								100%		
62	fetish								89%		11%
63	bar								100%		
64	protect				11%				78%		11%
65	article								82%		18%
66	homosexuality						100%				
67	bathhouse						100%				
68	heterosexual						100%				
69	evidence					6%	94%				

70	party						69%		14%		17%
71	lockdown						78%	22%			
72	human							100%			
73	transmission							100%			
74	activity			5%		15%		80%			
75	airborne				52%			48%			
76	high							80%	15%		5%
77	pandemic							100%			
78	blame				100%						
79	kill				100%						
80	die				100%						
81	quarantine				100%						
82	target		6%		80%			14%			
83	vaccine									100%	
84	stigma									100%	
85	stigmatize									100%	
86	african									100%	
87	reporting									100%	
88	std									100%	

89	fear						12%			83%	5%
90	close										100%
91	identify										100%
92	skin										100%
93	person										100%
